# Up‐regulated SAMD9L modulated by TLR2 and HIF‐1α as a promising biomarker in tuberculosis

**DOI:** 10.1111/jcmm.17307

**Published:** 2022-04-07

**Authors:** Xiang‐juan Zhang, Hai‐shan Xu, Chong‐hui Li, Yu‐rong Fu, Zheng‐jun Yi

**Affiliations:** ^1^ Department of Pathogen Biology School of Basic Medicine Weifang Medical University Weifang China; ^2^ School of Medical Laboratory Key Laboratory of Clinical Laboratory Diagnostics in Universities of Shandong Weifang Medical University Weifang China

**Keywords:** HIF‐1α, interferons, miR‐181b‐5p, *Mycobacterium tuberculosis*, SAMD9L, TLR2, TLR4

## Abstract

The aim of this study was to identify potential biomarkers of TB in blood and determine their function in Mtb‐infected macrophages. First of all, WGCNA was used to analyse 9451 genes with significant changes in TB patients’ whole blood. The 220 interferon‐γ‐related genes were identified, and then 30 key genes were screened using Cytoscape. Then, the AUC values of key genes were calculated to further narrow the gene range. Finally, we identified 9 genes from GSE19444. ROC analysis showed that SAMD9L, among 9 genes, had a high diagnostic value (AUC = 0.925) and a differential diagnostic value (AUC>0.865). To further narrow down the range of DEGs, the top 10 hub‐connecting genes were screened from monocytes (GSE19443). Finally, we obtained 4 genes (SAMD9L, GBP1, GBP5 and STAT1) by intersections of genes from monocytes and whole blood. Among them, it was found that the function of SAMD9L was unknown after data review, so this paper studied this gene. Our results showed that SAMD9L is up‐regulated and suppresses cell necrosis, and might be regulated by TLR2 and HIF‐1α during Mtb infection. In addition, miR‐181b‐5p is significantly up‐regulated in the peripheral blood plasma of tuberculosis patients, which has a high diagnostic value (AUC = 0.969).

## 1 INTRODUCTION

Tuberculosis (TB), caused by the bacillus *Mycobacterium tuberculosis* (Mtb), is an important public health problem worldwide. A person only needs to breathe in a small number of bacteria to become infected. Globally, an estimated 10 million TB people fell ill with TB in 2019.^1^ Mtb is a microbe that is particularly successful at surviving within macrophages. Its success depends on transforming antibacterial cells into their habitats and vehicles of transmission, which rely on the complex immune escape mechanism. It inhibits the maturation and acidification of phagolysosomes, resists the damage of free radicals, inhibits oxidative stress and function of reactive oxygen and reactive nitrogen intermediates, affects the way of cell death (inhibits apoptosis and autophagy). These mechanisms make the Mtb persist in macrophages, evade the body's immune system, and create favourable conditions for bacteria breeding and spreading.^2^, ^3^, ^4^

Toll‐like receptors (TLR) as pattern recognition receptors (PRR) can recognize multiple PAMP on the surface of Mtb. There is much literature showing that a variety of TLRs are involved in the entry process of Mtb and are closely related to the outcome of Mtb after entry.^5^, ^6^, ^7^ Among them, TLR2 and TLR4 deserve special attention.^5^, ^8^, ^9^

The aim of this study was to identify potential biomarkers of tuberculosis in blood and determine their function in Mtb infected macrophages. Bioinformatics methods were used to screen out genes that played a significant role in Mtb infection from the whole blood sample. In order to further narrow the range, four core genes were obtained by intersection with genes that played a significant role in monocytes. Subsequently, relevant experiments were designed to explore the function and upstream molecules of key genes. This will provide new ideas for the diagnosis and treatment of TB.

## 2 MATERIALS AND METHODS

### 2.1 Hub gene screening cohort

To select potential biomarkers, we first analysed whole blood samples (GSE19444, select 12 HC and 21 ATB) to obtain gene modules that were significantly changed in TB by WGCNA.^10^ We then construct the protein–protein interaction (PPI) network of key genes in this module. Then Cytohubba plug‐in in Cytoscape software was used to narrow the gene range further. AUC values of key genes were calculated to further screen key genes with diagnostic values.

In order to further narrow the range of key genes, and obtain differentially expressed genes (DEGs) with the value of mechanism research. We used GEO 2R and Cytohubba plug‐in to screen out key genes from the monocyte database (GSE19443, select 4 HC and 7 ATB).

Taking the intersection of key genes from whole blood and monocytes, we end up with genes that have both diagnostic value and mechanistic value.

### 2.2 Hub gene verification cohort

Whole blood samples are easy to obtain clinically, so they are suitable for screening diagnostic markers. In order to further explore the differential diagnostic value of key genes screened from whole blood, we extracted the expression data of key genes from GSE 42826 database, drew scatter plots and ROC curves, and calculated their AUC values.

In clinical treatment, how to evaluate the effectiveness of treatment measures, so appropriate monitoring indicators are needed. To this end, we further explore the changes of these genes during treatment, hoping to monitor their expression levels to evaluate whether treatment measures are appropriate and prevent the occurrence of undertreatment and overtreatment. The changes of expression levels of key genes in the database during treatment (GSE19435) were extracted, and the scatter diagram and broken line diagram were drawn.

### 2.3 Hub gene functional study

#### 2.3.1 Expression levels of key genes were detected

RAW264.7 were obtained from Stem Cell Bank, Chinese Academic of Sciences. Mouse BMDMs were isolated from tibia and femur of C57BL/6 mice which were purchased from Medical Laboratory Animal Center of Weifang Medical University and differentiated into macrophage by 30 ng/mL murine recombinant macrophage colony‐stimulating factor (M‐CSF) (PEPROTECH, USA) treatment for 5 days. When cells grew vigorous, we discarded a part of medium, added the bacterial liquid in proportion. After 6h, fresh medium was replaced, which was marked as infection 0.

Total RNA from cells and tissue was extracted by Trizol (Life Technologies). The purity and concentration of RNA were then measured by K5800 differential photometer (KAIAO). The reverse transcriptional condition of the final gene is 42℃ for 60 min, then 95℃ for 5 min. The amplification condition is 94℃ for 30s, 61℃ for 20s and 72℃ for 20s. The amplification of miRNA is 94℃ for 15s, 60℃ for 25s, 72℃ for 10s. The annealing temperature of tubulin and U6 is 61℃ and 60℃.

#### 2.3.2 Functional exploration of key genes in Mtb infection

Three siRNA for the final gene were synthesized by GenePharma. The sense and antisense strands of SAMD9L siRNA1 are 5'‐GCC GCU GUU UCA AGA AAU ATT‐3' and 5'‐UAU UUC UUG AAA CAG CGG CTT‐3'. The sense and antisense strands of SAMD9L siRNA2 are 5'‐GCA CCU AAG AAU UCU UAU ATT‐3' and 5'‐UAU AAG AAU UCU UAG GUG CTT‐3'. The sense and antisense strands of SAMD9L siRNA3 are 5'‐GUC CAG CAC UUC UGA UAA ATT‐3' and 5'‐UUU AUC AGA AGU GCU GGA CTT‐3'. The sense and antisense strands of stable negative control (NC) are 5'‐UUC UCC GAA CGU GUC ACG UTT‐3' and 5'‐ACG UGA CAC GUU CGG AGA ATT‐3'. After transfection with Lip2000 (Thermo Fisher Scientific), we screen out the one with the most interference effect for subsequent experiments.

After transfection about 24 h, Mtb infection was carried out at a ratio of 1:10. Twenty‐four hours after infection, we discarded the supernatant and 200 μLPBS was added, followed by 5 μL AO and 5 μL EB staining solution. After mixing, the cells were incubated for 5 min and kept in dark place. And then, the supernatant was discarded and 500 μL PBS which containing 25 μL Hoechst and 25 μL PI staining solution was added. After mixing, the cells were incubated for 30 min, and then washed twice with PBS. Next, fluorescence microscope was used to observe. Finally, acid‐fast staining was performed on the cell slide, and the number of acid‐fast bacilli in 100 cells was counted under the oil microscope.

#### 2.3.3 To explore the effect of upstream genes on key genes

In order to explore the regulatory role of upstream genes on key genes, we interfered with TLR2 of RAW264.7 cells, then infected with Mtb, and subsequently measured the expression level of SAMD9L. TLR4 in the same way.

The sense and antisense strands of TLR2 siRNA are 5'‐CCC AAA GUC UAA AGU CGA UTT‐3' and 5'‐AUC GAC UUU AGA CUU UGG GTT‐3'. The sense and antisense strands of TLR4 siRNA are 5'‐CCU AGU ACA UGU GGA UCU UTT‐3' and 5'‐AAG AUC CAC AUG UAC UAG GTT‐3'.

In addition, to investigate whether there is a regulatory relationship between HIF‐1α and key genes, we added HIF‐1α inhibitor into the medium, co‐incubated with RAW264.7 cells for 24 h, and then infected Mtb. Subsequently, the expression level of SAMD9L was determined.

### 2.4 miRNAs prediction of key hub gene and validation

miRWalk, TargetScan and StarBase were used to predict the miRNAs which may target key hub genes. Then, we take the intersection of the prediction results of the three databases and draw a Venn diagram. We selected the database GSE131708 (PBMC, 4 TB meningitis patients, 4 HC) to verify the reliability of the predicted TB miRNAs.

### 2.5 Clinical samples were collected and quantified by quantitative reverse transcription‐polymerase chain reaction (qRT‐PCR)

A total of 16 plasma samples (8 TB and 8 HC) were collected from Weifang Second People's Hospital. The inclusion criteria for active tuberculosis are as follows: (1) at least one positive bacteriological test (including sputum smear and culture process); (2) Chest X‐ray examination revealed obvious active tuberculosis foci; (3) Histopathological examination confirmed tuberculosis; (4) Suspected tuberculosis patients with similar clinical symptoms were proved to be significantly improved after clinical anti‐tuberculosis treatment. Tuberculosis patients who did not receive anti‐tuberculosis treatment at initial diagnosis. Patients with co‐existing cancer, diabetes, autoimmune disease, hypertension, chronic inflammation and pregnant or lactating women were excluded from the active TB group. Informed consent was obtained from all patients prior to beginning the study.

Total RNA was extracted from each sample using Trizol‐LS (Life Technologies, USA) according to the manufacturer's instruction. Purified RNA was reverse transcribed to cDNA using Prime‐ScriptH RT reagent Kit (TaKaRa) according to the manufacturer's protocol. qRT‐PCR was then performed using SYBRTM Green PCR Master Mix (Takara) following standard conditions on LightCycler 480 Ⅱ real‐time PCR system (Roche). The relative amount of expressed miRNA was calculated by comparison between case and control using the 2^−ΔCt^ method.

## 3 RESULTS

### 3.1 Dataset preprocessing and key gene screening

#### 3.1.1 The preliminary screening (GSE19444, whole blood samples)

**FIGURE 1:**
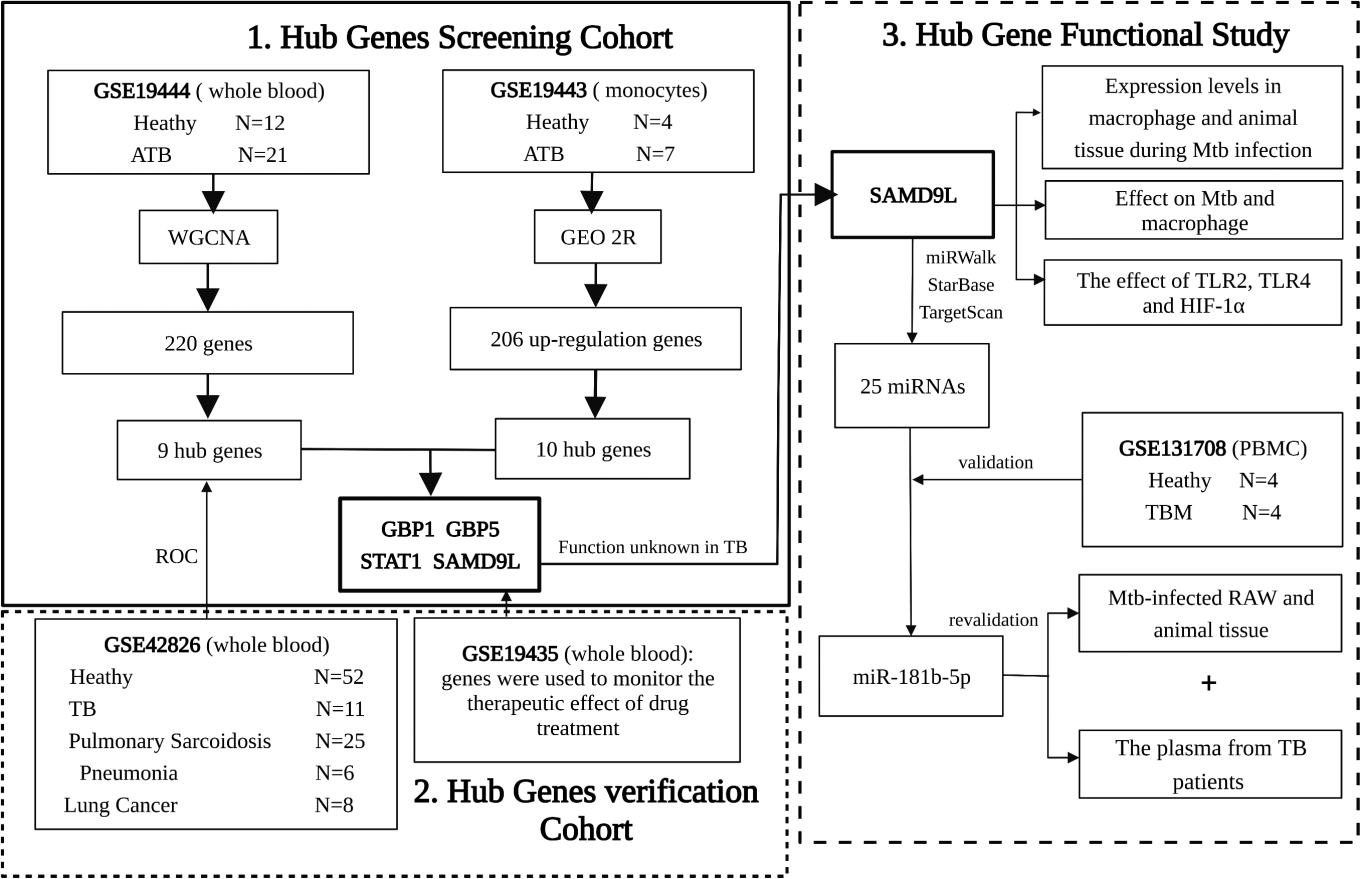
Flowchart of microarray analysis of monocytes and whole blood samples

**FIGURE 2:**
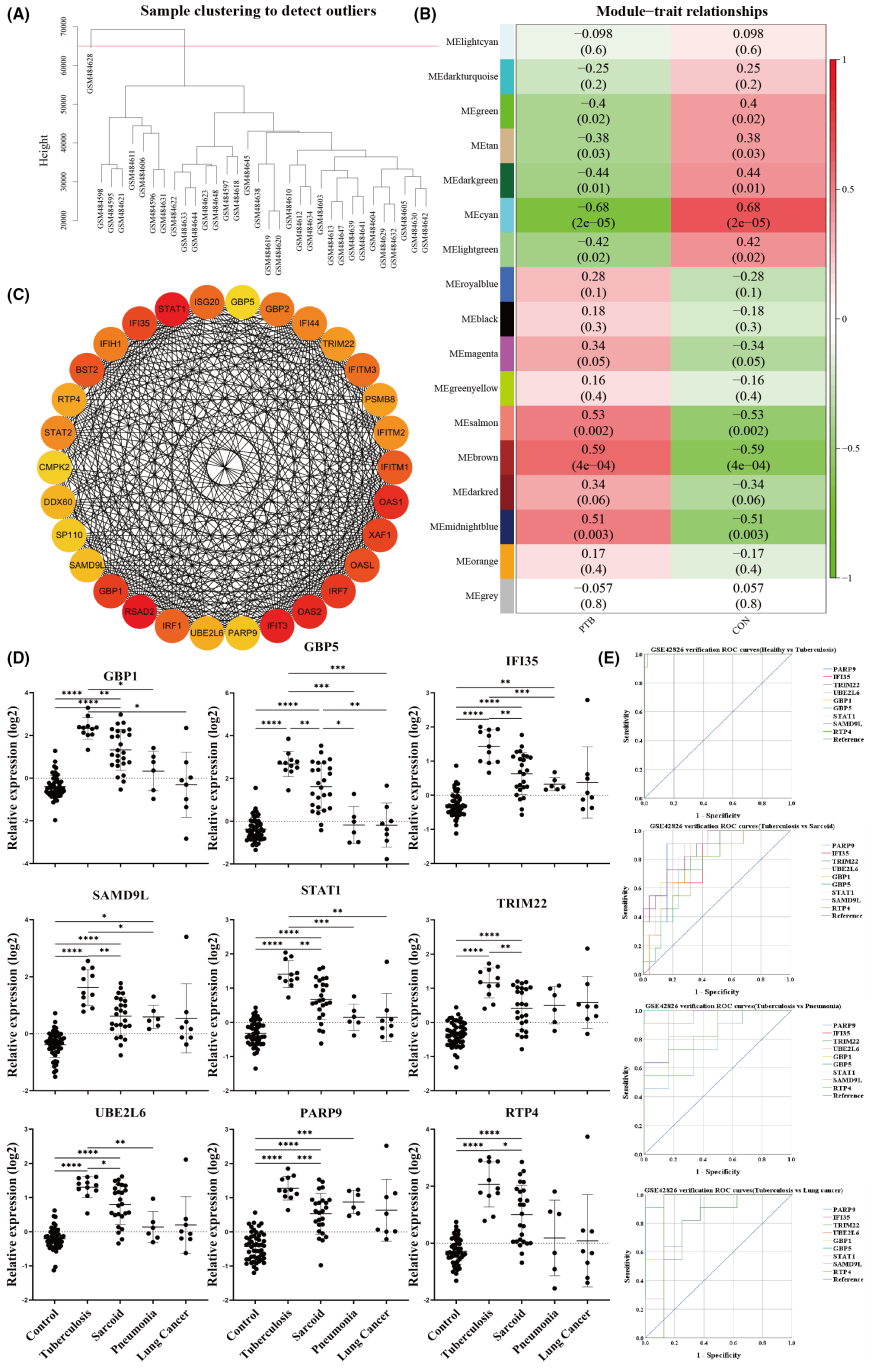
Identification of significant modules, enrichment of biological process and hub genes screening by WGCNA from whole blood samples. (A) Sample clustering of GSE19444 to detect outliers. The cut height was set as 65000 with one deviated sample of GSM484628. (B) Module‐Trait relationship heat map. Each row corresponds to a colour module and column corresponds to a clinical trait (PTB and Control). The upper number in each cell refers to the correlation coefficient of each module in the trait, and the lower number is the corresponding P value. Among them, the brown module was the most relevant module with PTB. (C) The top 30 hub genes were identified by CytoHubba. (D) The expression of 9 genes in GSE42826. (E) The ROC curves of 9 genes in GSE42826

To screen out the key genes and the main biological processes that changed during TB, we downloaded gene expression matrix from GSE19444, selected 9451 genes with the highest expression variance in the top 25% after probe annotation (indicating high variability among different groups, including up‐ and down‐regulated genes), the gene expression was filtered with mean FPKM = 0.5 as the standard and 9439 genes remained. Subsequently, in order to build a stable co‐expression network, outlier sample GSM484628 was removed (Figure 1 and Figure 2A). Taking *R* ^2^ = 0.85 as the criterion to meet the scale‐free condition, we chose the optimal weighting parameter *β* = 9 (*R* ^2^ = 0.856). Not only ensured that the network was close to the scale‐free network and the minimum threshold, but also ensured that the average connection degree of the network was not too small, which was conducive to the network to contain enough information.

Furthermore, we searched gene modules closely associated with TB. The brown (correlation index = 0.59, *p* = 4E‐04) module (including 1701 genes) had the highest positive correlation with PTB (Figure 2B). Meanwhile, the correlation coefficient between GS and MM in the brown module (cor = 0.76, P<1E‐200) further verify the reliability of the results. Thus, we thought that genes in the brown module play an important role in PTB.

The inclusion criteria for important genes in brown module was set as follows: GS>0.30 and MM>0.80. Then we got 220 important genes and uploaded it to DAVID. We can see type Ⅰ interferon play a significant role in whole blood (Table 1). Simultaneously, 220 genes were uploaded to STRING and the results were imported into Cytoscape. According to node degree, the top 30 hub genes were identified by CytoHubba (Figure 2C).

**TABLE 1:** Top 10 of the BP analysis of 220 important genes in GSE19444

Term	Count	*p* value	Genes
Response to interferon‐beta	6	4.54E−09	PLSCR1, BST2, IFITM1, IFITM2, IFITM3, XAF1
Response to interferon‐gamma	7	2.81E−08	BST2, IFITM1, IFITM2, IFITM3, CALCOCO2, TRIM21, GCH1
Defence response to virus	11	9.38E−08	IFIT3, TRIM5, PLSCR1, OASL, BST2, DDX60, IRF1, RSAD2, OAS1, OAS2
Negative regulation of viral genome replication	7	9.59E−07	PLSCR1, OASL, BST2, IFITM1, IFITM2, IFITM3, RSAD2
Positive regulation of I‐kappaB kinase/NF‐kappaB signalling	10	2.39E−05	TRIM5, SECTM1, TNFSF10, BST2, TICAM2, RBCK1, RIPK2, TLR6, CASP1, TRIM22
Response to interferon‐alpha	4	2.52E−04	BST2, IFITM1, IFITM2, IFITM3
Innate immune response	10	2.59E−04	IFIT3, TRIM5, IFIH1, BST2, RELB, RIPK2, JAK2, CLEC4D, MR1, TLR6
Intracellular transport of viral protein in host cell	3	4.17E−04	TAP2, TAP1, DYNLT1
Regulation of MyD88‐dependent toll‐like receptor signalling pathway	3	4.17E−04	IRF7, IRF1
Cellular response to interferon‐beta	4	5.42E−04	IRF1, STAT1, AIM2

To further screen the essential genes that can distinguish TB patients from HC, expression levels of 30 hub genes were extracted from GSE19444 (Table 2). SPSS was used to calculate the AUC, and we set up AUC>0.9, log_2_FC>1.0 as filter criteria to narrow the range of hub genes down, finally obtained 9 essential genes: PARP9, SAMD9L, RTP4, IFI35, TRIM22, STAT1, GBP5, GBP1 and UBE2L6.

**TABLE 2:** Extract the expression levels of 30 hub genes in GSE19444, and calculate their logFC and AUC values

GEO database	No.	Gene symbol	Screening index		GEO database	No.	Gene symbol	Screening index	
			logFC	AUC				logFC	AUC
GSE19444	1	IFITM1	0.9194845	0.956	GSE19444	16	IRF7	0.9229076	0.885
	2	IRF1	0.6982569	0.948		17	IFITM3	1.7851673	0.881
	3	PARP9	1.19087565	0.944		18	IFIH1	0.8990314	0.877
	4	SAMD9L	1.1106799	0.925		19	STAT2	0.7607976	0.873
	5	RTP4	1.5196945	0.921		20	IFIT3	1.5926177	0.865
	6	PSMB8	0.6366932	0.917		21	OAS1	1.2617923	0.841
	7	IFI35	1.1601648	0.913		22	RSAD2	1.7587783	0.837
	8	GBP2	0.9730581	0.913		23	OASL	1.20553685	0.829
	9	IFITM2	0.6037955	0.913		24	XAF1	1.1103368	0.825
	10	SP110	0.5398558	0.913		25	BST2	0.5583127	0.821
	11	TRIM22	1.1750444	0.909		26	IFI44	1.2609401	0.817
	12	STAT1	1.1416413	0.909		27	DDX60	0.8492459	0.817
	13	GBP5	2.2409449	0.905		28	OAS2	0.8256228	0.794
	14	GBP1	1.92857825	0.901		29	CMPK2	0.9911882	0.782
	15	UBE2L6	1.13141065	0.901		30	ISG20	0.4403553	0.76

#### 3.1.2 Further narrow down the number of key genes (GSE19443, monocytes from whole blood)

According to the sample information and gene expression matrix of GSE19443, we used GEO 2R to screen 320 DEGs, among which, 206 were up‐regulated genes and 114 were down‐regulated. The screening criteria for DEGs were as follows: *p* < 0.01 and log_2_FoldChange>1 is the up‐regulated gene; *p* < 0.01 and log_2_FoldChange<−1 is the down‐regulated gene.

To analyse the function of DEGs, GSEA, DAVID and Fun Rich were used for gene enrichment analysis of samples.

**TABLE 3:** AUC of 9 genes in healthy people, tuberculosis, sarcoidosis, pneumonia and lung cancer

Multiple comparisons	AUC^†^								
(GSE42826)	GBP1	GBP5	STAT1	SAMD9L	PARP9	IFI35	TRIM22	UBE2L6	RTP4
Control vs. Tuberculosis	1.00	0.998	1.00	1.00	1.00	0.998	0.998	0.998	1.00
Tuberculosis vs. Sarcoidosis	0.822	0.778	0.855	0.865	0.873	0.822	0.833	0.771	0.789
Tuberculosis vs. Pneumonia	0.985	1.00	0.985	0.924	0.773	0.985	0.864	0.985	0.864
Tuberculosis vs. Lung Cancer	0.943	0.989	0.909	0.875	0.807	0.875	0.773	0.875	0.875

^†^AUC, area under curve.

**FIGURE 3:**
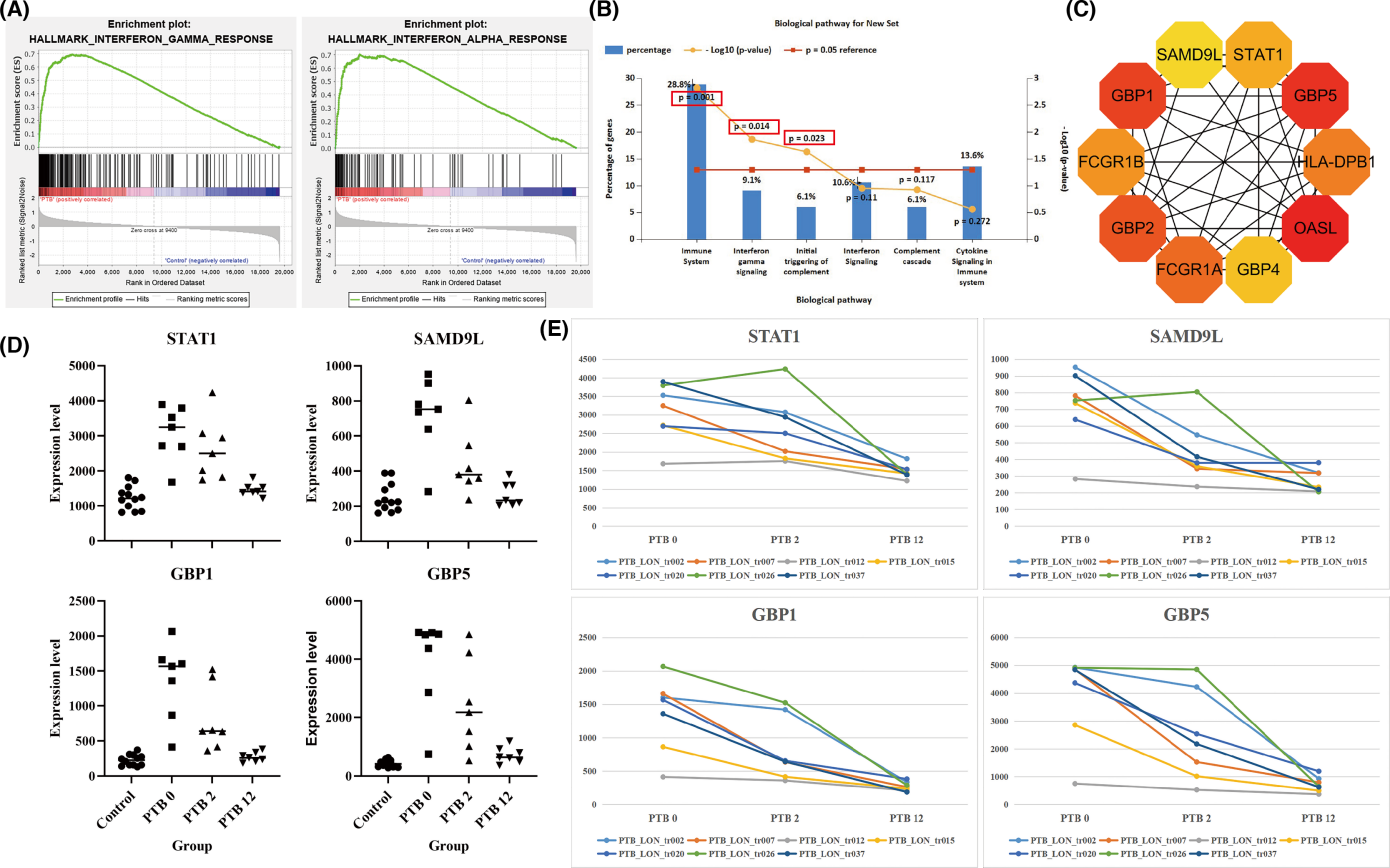
DEGs screening, enrichment of biological process and identification of hub gene in monocyte. (A) Gene set enrichment analysis (GSEA) using GSE19443. h.all.v6.2.symbols.gmt [Hallmarks] gene set database was used to analyse the expression data of the TB and HC samples. The screening criteria for significantly enriched gene sets were NES>1, NOM *p*‐val<0.05, FDR q‐val<0.25. Only listed the two most common functional gene sets enriched in PTB monocytes samples with up‐regulated DEGs. (B) The Funrich software drew a bar charts of 6 biological pathways according to the *p* value and the percentage of genes, among which biological pathways with *p* < 0.05 was significant. (C) According to node degree, the top 10 hub genes were identified by CytoHubba. The darker the colour, the higher the score. (D) (E) Scatter and line plots of 4 genes. The samples’ group: Control (healthy controls); PTB 0 (pre‐treatment); PTB 2 (2 months after treatment initiation); PTB 12 (12 months after treatment initiation)

The GSEA enrichment analysis of gene sets in TB revealed that the gene sets were significantly enriched in interferon‐γ response (Figure 3A). DAVID and Funrich were used to analyse 206 up‐regulation genes in TB. GO enrichment analysis indicated that the enrichment of up‐regulated DEGs were mainly related to interferon‐γ‐mediated signalling pathway in BP (Table 4). A total of 206 up‐regulated DEGs were uploaded to Funrich and analysed for biological pathways of PTB samples. The results showed that up‐regulated DEGs were mainly enriched in the immune system, interferon‐γ signalling, and initial triggering of complement (Figure 3B).

**TABLE 4:** BP analysis of 206 up‐regulation genes in GSE19443

Term	Count	*p* value	Genes
interferon‐gamma‐mediated signalling pathway	8	3.55E−06	OASL, FCGR1A, FCGR1B, CAMK2D, HLA‐DPB1, STAT1, GBP2, GBP1
antigen processing and presentation of endogenous peptide antigen via MHC class I	3	0.0017	TAP2, TAP1, TAPBP
response to hypoxia	7	0.0048	CD38, SCFD1, MYOCD, PLOD2, CLDN3, CAMK2D, BAD
blood circulation	4	0.0078	LPA, EPB41, SERPING1, STAT1
cytosol to ER transport	2	0.0180	TAP2, TAP1
cellular response to drug	4	0.0247	REST, GAS6, DPEP1, AIM2
antigen processing and presentation of peptide antigen via MHC class I	3	0.0300	TAP2, TAP1, TAPBP
signal transduction	18	0.0337	NAMPT, CLDN3, PPP1R12B, RASSF9, MAPK11, NR2E3, FGF20, ARRDC2, GAS6, CD38, LGALS3BP, GRB10, WISP2, IL12RB1, FCGR1A, CSF2RB, GNB3, TAAR5

Combined with the above results, we can know that the interferon‐γ‐related signalling pathway is up‐regulated in monocyte during Mtb infection.

To screen out the essential up‐regulated DEGs, we uploaded 206 up‐regulated DEGs to STRING for further analysis. The PPI network was analysed by Cytoscape. According to node degree, the top 10 hub genes were identified by CytoHubba (Figure 3C).

#### 3.1.3 Final differential gene identification

The analysis results of GSE19444 and GSE42826, we got 9 genes with diagnostic value: PARP9, SAMD9L, RTP4, IFI35, TRIM22, STAT1, GBP5, GBP1 and UBE2L6; after analysing GSE19443, we got 10 genes with significant functions: SAMD9L, GBP1, GBP2, GBP4, GBP5, FGCR1A, FGCR1B, OASL, HLA‐DPB1 and STAT1. Finally, we obtained 4 genes by intersections of genes from monocyte and whole blood: SAMD9L, GBP1, GBP5 and STAT1.

### 3.2 Evaluation of differential diagnostic ability

Whole blood samples are easy to obtain, easy to handle and more suitable for diagnosis. So, to further evaluate differential diagnosis of these 9 gene in TB, sarcoidosis, pneumonia and lung cancer, we extracted their expression data from GSE42826 for comparison and drew ROC curves. The relative expression levels of these 9 genes in TB were higher than in another group (Figure 2D).

Moreover, we drew ROC curves and calculated AUC of the 9 genes. The AUC of 9 genes were above 0.7, respectively, showing good diagnostic performance in differentiating TB patients from HC and another disease (Figure 2E, Table 3).

In addition, to further evaluate the monitoring ability of 4 genes in drug therapy for TB patients, we extracted the expression data of 4 genes in GSE19435, and drew scatter plots and broken line plots (Figure 3D,E). Blood was taken from the control, pre‐treatment (PTB 0), 2 months after treatment initiation (PTB 2), and 12 months after treatment initiation (PTB 12), which would be after treatment was completed. With the prolonging of treatment, the expression levels of 4 genes decreased gradually and dropped to the level of control after 12 months of treatment.

### 3.3 Hub gene functional study

#### 3.3.1 SAMD9L up‐regulated in RAW264.7 and BMDM during Mtb infection

**FIGURE 4:**
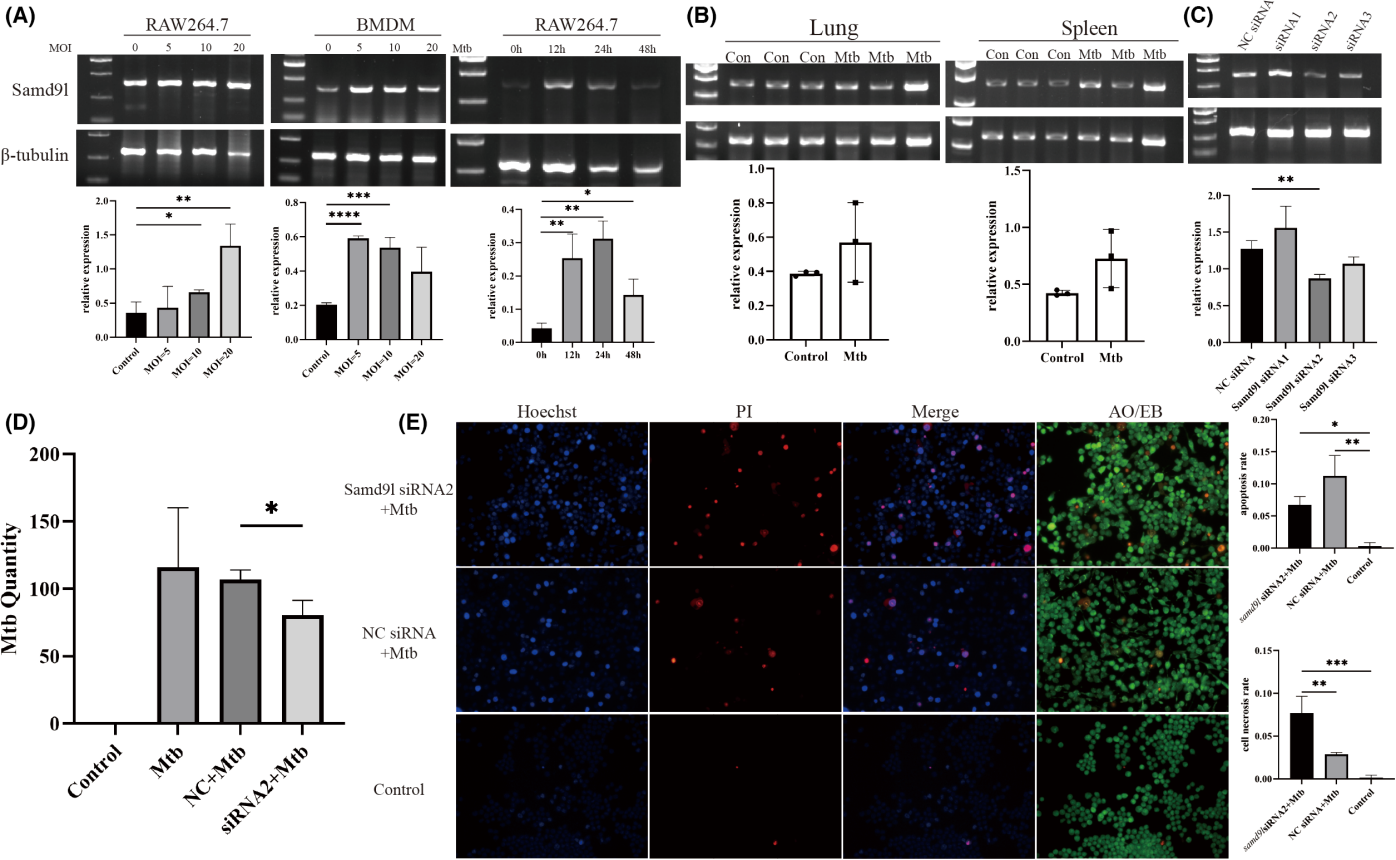
(A) Expression of SAMD9L in RAW264.7 and BMDM after Mtb infection. (B) The expression of SAMD9L in the lung and spleen from healthy and TB mice. (C) siRNA2 shows good interferon effect of SAMD9L. (D) Effects of SAMD9L on Mtb. (E) Hoechst/PI/AO/EB staining. *: *p* < 0.05, **: *p* < 0.01, ***: *p* < 0.001

Based on the above bioinformatics analysis results and literature review, we found that the function of SAMD9L was unknown, so we want to further explore its expression in RAW264.7 and BMDM, and explore its effect in RAW264.7 during Mtb infection. Our results showed that SAMD9L was up‐regulated in RAW264.7 and BMDM after Mtb infection, and its expression reached the highest level after 24 h of infection (Figure 4A). In order to comprehensively explore the expression level of SAMD9L after Mtb infection, we constructed a mouse model of Mtb infection and measured the expression level of lung and spleen tissues after infection. Finally, we found that the expression of SAMD9L increased in the spleen and lungs of infected mice (Figure 4B).

#### 3.3.2 SAMD9L can inhibit cell necrosis, which is not conducive to Mtb survival during Mtb infection

siRNA with the most significant interference effect was screened (Figure 4C). After cell transfection, the cells were infected for 24 h and stained for observation. Next, AO/EB and Hoechst/PI staining were used to test the effects of SAMD9L in RAW264.7. We found that compared with NC‐siRNA, necrosis was significantly increased after SAMD9L inhibition. This suggests that SAMD9L may not conducive to the intracellular survival of Mtb.

#### 3.3.3 The effect of TLR2, TLR4 and HIF‐1α

**FIGURE 5:**
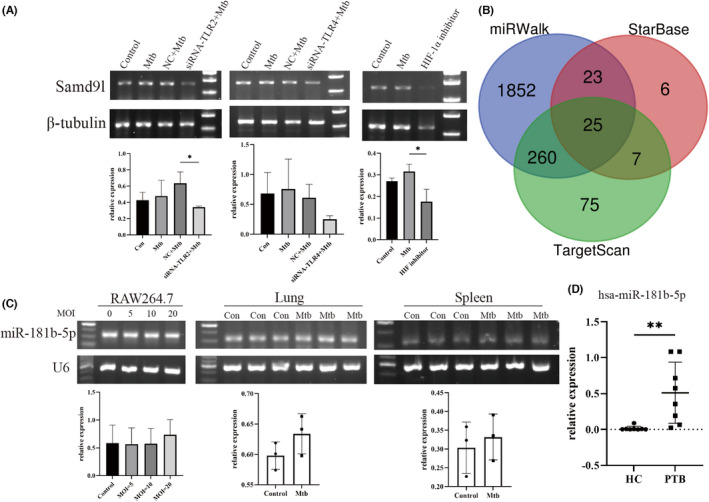
(A) After interferes the expression of TLR2 and TLR4 and inhibits HIF‐1α, the expression of SAMD9L. (B) Predicted miRNAs and their validation. The number of predicted miRNAs by the miRWalk, StarBase and TargetScan. (C) The expression of miR‐181b‐5p in RAW264.7 and Mtb infected mice. (D) Expression of miR‐181b‐5p in plasma of TB patients and healthy controls

TLRs and HIF‐1α play an important role in Mtb infection. To further prove whether there is a regulatory relationship between TLR2, TLR4 and HIF‐1α, we interfered with TLR2 and TLR4 and inhibited HIF‐1α, respectively, followed by Mtb infection. We found that the expression of SAMD9L was significantly decreased after interference with TLR2 and inhibition of HIF‐1α. This implies that TLR2 and HIF‐1α regulate SAMD9L. However, after TLR4 interference, the expression of SAMD9L only decreased. (Figure 5A).

### 3.4 miRNAs prediction of key hub gene and validation

#### 3.4.1 miRNAs prediction and validation

miRNA is non‐coding small molecule that can play an essential role by suppressing gene expression. We tried to find miRNA that could interact with SAMD9L. To make the prediction more credible, we used StarBase, Targetscan and miRWalk to predict it. We got 25 miRNAs after intersecting of the results (Figure 5B). We selected GSE131708 dataset to verify the reliability of the above miRNAs in TB. Finally, we found that compared with HC, miR‐146a‐5p and miR‐181b‐5p were significantly reduced in TB.

#### 3.4.2 miRNA revalidation in Mtb‐infected macrophage and plasma from TB patients

Based on previous research by others, miR‐146a‐5p was proved to increase significantly in Mtb‐infected RAW264.7 and exosomes from it.^11^ Therefore, we chose to measure the expression of miR‐181b‐5p. Although the expression of miR‐181b‐5p was not statistically significant, it showed an upward trend after infection in RAW264.7 and tissue (Figure 5C).

What is the expression level of miR‐181b‐5p in the plasma of TB patients? To explore this question, we collected plasma from 8 HC and 8 TB patients, then has‐miR‐181b‐5p was quantitatively analysed by qRT‐PCR. The results showed that the expression of miR‐181b‐5p was a significant increase in the plasma from TB patients (Figure 5D).

Furthermore, we also calculated the AUC value, sensitivity and specificity were 0.969, 100%, 87.5% (95%CI: 0.892–1.000). Specificity and sensitivity meet FIND/WHO target products profiles (TPPs).^12^, ^13^, ^14^ This means that miR‐181b‐5p could be a good clinical diagnostic biomarker in TB.

## 4 DISCUSSION

TB represents an important public health problem worldwide. Until the COVID‐19 pandemic, TB was the leading cause of death from a single infectious agent, ranking above HIV/AIDS.^15^ Therefore, there is an urgent need to accelerate research on the pathogenesis of TB in order to improve diagnosis and treatment methods, shorten the time of diagnosis and treatment, and effectively reduce the spread of the disease.

WGCNA was applied to the blood transcriptional data from GSE19444, modules of co‐expressed genes with a coherent functional relationship were generated. The range of genes that we selected to analysis is different from the research of Singhania A et al,^16^ but there was a great similarity in the composition of the blood transcript signatures, with an overabundance of IFN‐inducible genes, in keeping with previous findings using microarray and RNA‐Seq based signatures.

Analysis results of expression profile from whole blood and monocyte show that two kinds of different status of interferon, in other words, type Ⅱ interferon play a significant role in monocyte, but it did not occupy the leading position in whole blood, we consider because it is affected by the number of monocytes in whole blood, and whole blood reflects the outcome of combined action of different cell types at the same time. We screened out 4 genes from monocyte and whole blood: SAMD9L, GBP1, GBP5 and STAT1. Included in 393 transcription profiles of active TB screened in the original text.^17^

We intersected critical genes from the whole blood and monocyte and found that the SAMD9L may play a role in TB. SAMD9L is a type Ⅰ interferon‐induced gene that has important roles during virus infection and innate immunity. Its role in the control of influenza virus, poxvirus infection, and also pathogenesis has been proposed.^18^, ^19^, ^20^ We searched for SAMD9L related pathways through KEGG but found no pathway information. Therefore, the function of SAMD9L in tuberculosis was preliminarily explored in this paper. First, we demonstrated that this gene is up‐regulated in Mtb infected macrophages, as predicted. The lung and spleen of tuberculosis infected mice showed a trend of up‐regulated expression, but there was no significant statistical significance. We believed that on the one hand, the gene expression difference was caused by individual differences of mice, and on the other hand, it might be the result of the combined effect of other kinds of cells in the lung and spleen.

Next, to verify the effects of SAMD9L on bacteria and cells in Mtb infection, we screened siRNA suitable for SAMD9L. After interfering the expression of SAMD9L, we found that cell necrosis significantly increased after Mtb infection, so SAMD9L may play a role in inhibiting cell necrosis during Mtb infection. However, after bacterial counting, it was found that compared with the NC+Mtb group, the number of bacteria in the siRNA2+Mtb group was significantly reduced. However, cell necrosis was beneficial to the survival of Mtb. We believed that this was due to the increase of cell necrosis and the increase of bacteria released into the supernatant, resulting in the decrease of intracellular bacteria.

Mtb basically invades airframe via respiratory tract. When the body is infected with Mtb, TLRs located on the membrane of macrophages and dendritic cells specifically recognize PAMP on tuberculosis. Subsequently, the transfer protein MyD88 is activated to recruit effector molecules such as IRAK, TRAF6, MAPKs and transcription growth factor β ‐associated protein kinase 1 to activate multiple intracellular signalling pathways, and jointly induce the activation of NF‐κB into the nucleus. The transcriptional expression of several downstream proinflammatory factors, chemokines, adhesion molecules and receptor molecular target genes is subsequently promoted.^5^, ^6^, ^7^ TLR2 and TLR4 play a leading role in sensing the invasion of pathogens and inducing further inflammatory responses after initiating natural immunity.^9^, ^21^

Therefore, in order to explore whether there is a regulatory relationship between SAMD9L and TLR2 and TLR4 molecules, we interfered with TLR2 and TLR4 respectively to conduct Mtb infection, and then measured the expression level of SAMD9L. We found that after TLR2 interference, the expression of SAMD9L decreased significantly, while after TLR4 interference, SAMD9L only decreased. Therefore, we believe that TLR2 can positively regulate SAMD9L, and there may be some molecules between TLR4 and SAMD9L that affect the expression of SAMD9L.

**FIGURE 6:**
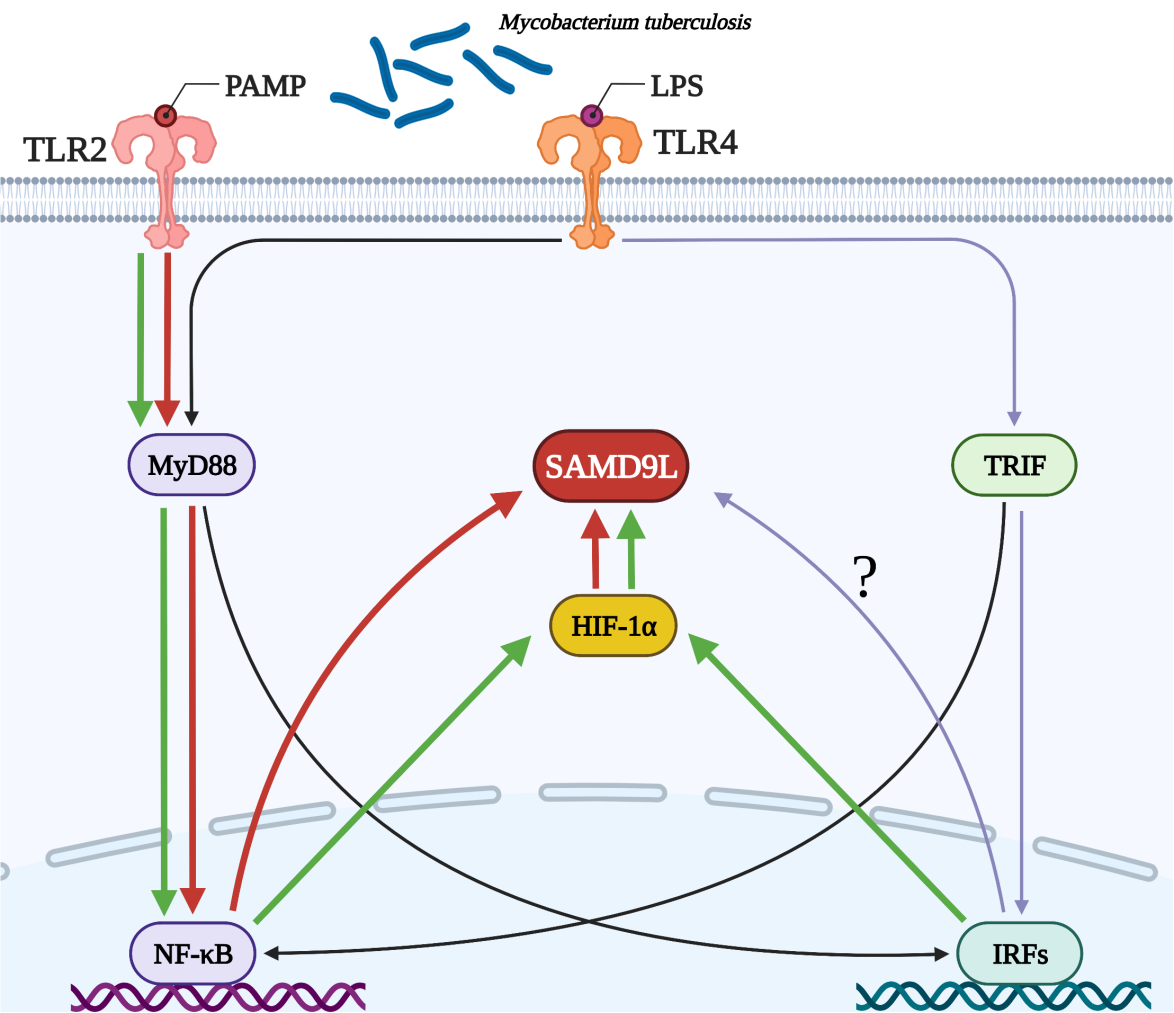
Intracellular mechanism pathway of SAMD9L

The transcription factor Hypoxia‐inducible factor‐1α (HIF‐1α) usually induces glycolysis gene expression under hypoxia conditions. Importantly, HIF‐1α is thought to be important in hypoxic environments or in innate immune responses to infection.^21^ In experimental studies, we found that the expression of SAMD9L was significantly decreased after inhibition of HIF‐1α, so HIF‐1α can regulate the expression of SAMD9L. It has been previously confirmed that TLR2 and TLR4 can regulate HIF‐1 α expression. In conclusion, TLR2 can regulate SAMD9L through HIF‐1α. However, there may be other molecules between TLR4 and SAMD9L that affect its expression (Figure 6).

miR‐181b‐5p only showed an upward trend in cells and animal tissues, while it was significantly up‐regulated in plasma of TB patients, so we believed that there was no direct regulatory relationship between miR‐181b‐5p and SAMD9L. However, miR‐181b‐5p can be a good diagnostic marker for TB.

In conclusion, we found that SAMD9L was significantly up‐regulated in whole blood of TB patients, monocytes in whole blood, Mtb infected RAW264.7 cells, and BMDM, and showed an upward trend in lung and spleen of Mtb infected mice. SAMD9L has good diagnostic value and differential diagnostic value. SAMD9L inhibits cell necrosis in Mtb infection. In Mtb infection, TLR2 and HIF‐1α regulate the expression of SAMD9L. In addition, although the experiment verified that there was no interaction between miR‐181b‐5p and SAMD9L, miR‐181b‐5p can be used as a good diagnostic marker for TB.

## CONFLICTS OF INTERESTS

All the authors declare that there are no conflicts of interest relevant to this article.

## AUTHOR CONTRIBUTION

**Xiang‐juan Zhang:** Conceptualization (equal); Data curation (equal); Formal analysis (equal); Methodology (equal); Software (equal); Visualization (equal); Writing – original draft (equal). **Hai‐shan Xu:** Data curation (equal); Formal analysis (equal). **Chonghui Li:** Data curation (equal); Formal analysis (equal). **yurong fu:** Conceptualization (equal); Methodology (equal); Writing – review & editing (equal); Funding acquisition (equal). **zhengjun yi:** Conceptualization (equal); Methodology (equal); Writing – review & editing (equal); Funding acquisition (equal).

## Data Availability

The datasets used for the current study are available from the corresponding author upon reasonable request.
